# Some Information Measures Properties of the GOS-Concomitants from the FGM Family

**DOI:** 10.3390/e24101361

**Published:** 2022-09-26

**Authors:** Florentina Suter, Ioana Cernat, Mihai Drăgan

**Affiliations:** 1 Faculty of Mathematics and Computer Science, University of Bucharest, Academiei 14, 010014 Bucharest, Romania; 2 “Gheorghe Mihoc-Caius Iacob” Institute of Mathematical Statistics and Applied Mathematics of Romanian Academy, Calea 13 Septembrie, 13, 050711 Bucharest, Romania

**Keywords:** concomitants, GOS, FGM family, Shannon entropy, Tsallis entropy, Awad entropy, residual entropy, past entropy, Fisher–Tsallis information number, Tsallis divergence

## Abstract

In this paper we recall, extend and compute some information measures for the concomitants of the generalized order statistics (GOS) from the Farlie–Gumbel–Morgenstern (FGM) family. We focus on two types of information measures: some related to Shannon entropy, and some related to Tsallis entropy. Among the information measures considered are residual and past entropies which are important in a reliability context.

## 1. Introduction

The notion of concomitants or induced order statistics arose in the early 1970s in the works of David [1] and Bhattacharya [2]. Briefly, when there is a sample from a bivariate distribution ordered by the first variate, the second variate paired with the r-th first variate is called the concomitant of the r-th-order statistic. Concomitants are important in situations in which are implied two characteristics and measuring one of them can influence the other. Therefore, they have applications in many fields such as selection procedures, inference problems, double sampling plans and systems reliability. For example, in [3,4] are studied from a reliability point of view complex systems with components which have two sub-components that performs different tasks, and in [5], the distribution theory of lifetimes of two component systems is discussed. In studies regarding the concomitants there are two elements that have to be mentioned: the kind of dependence between first and second variate, and the kind of order for the first variate. The majority of studies are based on the hypothesis of simple order statistics, but there are also studies that assume different kinds of orders such as as record values order or generalized order statistics.

Generalized order statistics (GOS) was introduced by Kamps [6] and it is a unifying concept for various types of order statistics such as simple order statistics, record values, sequential order statistics.

In this paper, we focus on the concomitants of GOS and with the dependence structure between the first variate and the second variate given by the Fairlie–Gumbel–Morgenstern (FGM) family. This family is a flexible family of bivariate distributions used as a modeling tool for bivariate data in many fields [7], one such field being Reliability, see [3,4,5]. The FGM family has a simple analytical form, but it can describe only relatively weak dependence because the correlation coefficient between the two components cannot exceed 1/3. To prevent this limitation, extensions of FGM family have been proposed, for example, iterative FGM distributions or Huang–Kotz FGM distributions [8,9,10,11,12]. The results obtained in our paper will be generalized for these extensions of FGM family in a future work.

For the concomitants mentioned above, we recall and determine properties that some information measures have. The information measures that we deal with are in two categories, information measures related to Shannon entropy and information measures related to Tsallis entropy.

Since it was introduced in physics and adapted to information theory by Shannon in 1948, the concept of entropy has become more and more important in fields such as information theory, code theory, probability and statistics, reliability.

In probability and statistics, entropy measures the uncertainty associated to a random variable. Taking as a starting point Shannon entropy, a series of entropies have been defined as a generalization of it. For the concomitants of GOS from the FGM family, we will look at Shannon-derived and Tsallis-derived entropies, and our main aim is to determine Awad-type extensions for all the considered entropies, because Awad entropies do not have several drawbacks that Shannon entropy, for example, has: different systems with the same entropy, possible negative values for continuous distributions, different results in discrete and continuous case of linear random variable transformation, etc.

Furthermore, for the concomitants of GOS from FGM, we will determine not only entropies, but also other information measures such as Tsallis divergence and shift-invariant Fisher–Tsallis information number.

In the following sections, we recall some definitions and properties of GOS and their concomitants, in particular, when the bivariate distribution is in the FGM family. Then, we will discuss Shannon-type entropies, Tsallis-type entropies, Fisher information and divergences for concomitants of GOS from the FGM family. For these concomitants, in the last section, we will introduce new extensions and results on information measures.

## 2. Generalized Order Statistics and Their Concomitants for the FGM Family

### 2.1. Generalized Order Statistics

The concept of GOS was introduced by Kamps [6], in 1995, who proposed an unifying pattern of various order statistics:

**Definition** **1.**  *The random variables $X(1,n,\tilde{m},k)$,…, $X(n,n,\tilde{m},k)$ are called GOS based on distribution function F with density function f, if their joint density function is given by:*

(1) $$f\left(x_{1},\ldots ,x_{n}\right)=k\left(\prod_{j=1}^{n-1}\gamma_{j}\right)\left(\prod_{i=1}^{n-1}{\left(1-F\left(x_{i}\right)\right)}^{m_{i}}f\left(x_{i}\right)\right){\left(1-F\left(x_{i}\right)\right)}^{k-1}f\left(x_{n}\right)$$

*on the cone $F^{-1}\left(0\right)<x_{1}\leq x_{2}\cdots \leq x_{n}<F^{-1}\left(1\right)$ of $R^{n}$ with parameters n∈N, n≥2, k>0, $\tilde{m}=\left(m_{1},\ldots ,m_{n-1}\right)$, $\gamma_{r}=k+n-r+\sum_{j=r}^{n-1}m_{j}>0$, for all r∈{1,2,…,n−1}.*

Some particular cases of GOS are:

- Simple order statistics with $m_{1}=m_{2}=\cdots =m_{n-1}=0$ and k=1;
- Common record values with $m_{1}=m_{2}=\cdots =m_{n-1}=-1$ and k=1;
- Sequential order statistics with $\gamma_{i}=\left(n-i+1\right)\alpha_{i}$, $\alpha_{1},\alpha_{2},\ldots ,\alpha_{n}>0$;
- Progressive type II censored order statistics based on censoring scheme $\left(R_{1},R_{2},\ldots ,R_{n}\right)$ with $\gamma_{n}=k=R_{n}+1$, $\gamma_{r}=n-r+1-\sum_{i=r}^{n}$, 1≤r≤n and $m_{r}=R_{r}$, 1≤r≤n, [13,14].

As a result of the complex formula of the joint density, finding the marginal distributions of (1) is a difficult task, but in some particular cases, marginal densities can be found. In [6], the marginal densities are determined for $m_{1}=m_{2}=\ldots m_{n-1}=m$, and in [15] for $\gamma_{1}\neq \gamma_{j}$, 1≤i≠j≤n. In the following, we will suppose that $m_{1}=m_{2}=\ldots m_{n-1}=m$, i.e., we are in the m-GOS case where simple order statistics, record values and progressive type II censored order statistics with equi-balanced censoring scheme are included. Now, the density (1) becomes:

(2) $$f\left(x_{1},\ldots ,x_{n}\right)=k\left(\prod_{j=1}^{n-1}\gamma_{j}\right)\left(\prod_{i=1}^{n-1}{\left(1-F\left(x_{i}\right)\right)}^{m}f\left(x_{i}\right)\right){\left(1-F\left(x_{i}\right)\right)}^{k-1}f\left(x_{n}\right)$$

on the cone $F^{-1}\left(0\right)<x_{1}\leq x_{2}\cdots \leq x_{n}<F^{-1}\left(1\right)$ of $R^{n}$ with parameters n∈N, n≥2, k>0, *m*, $\gamma_{r}=k+\left(n-r\right)\left(m+1\right)>0$, for all r∈{1,2,…,n−1}.

The marginal density function of the r-th GOS, r=1,2,…,n, in this case, is given by [6]:

(3) $$f_{r}\left(x\right)=\frac{c_{r-1}}{(r-1)!}{\left(1-F\left(x\right)\right)}^{\gamma_{r}-1}f\left(x\right)g_{m}^{r-1}\left(F\left(x\right)\right)$$

where $c_{r-1}=\prod_{j=1}^{r}\gamma_{j}$ and for x∈[0,1):

$$g_{m}\left(x\right)=\left\{\begin{array}{c} \frac{1}{m+1}\left(1-{\left(1-x\right)}^{m+1}\right),\;\;m\neq -1\\ \log\left(\frac{1}{1-x}\right)\;\;m=-1. \end{array}\right.$$

**Remark** **1.**  *For m=0 and k=1, i.e., the case of simple order statistics, we have $\gamma_{r}=n-r+1$, $c_{r-1}=\gamma_{1}\gamma_{2}\cdots \gamma_{r}=n\left(n-1\right)\ldots \left(n-r+1\right)$, $g_{0}\left(F\left(x\right)\right)=F\left(x\right)$, and (3) becomes the well-known marginal density of the r-th-order statistic.*

*For m=−1 and k=1, i.e., the case of record values, (3) becomes*

(4) $$f_{r}\left(x\right)=\frac{1}{(r-1)!}f\left(x\right){\left[-\ln\left(1-F\left(x\right)\right)\right]}^{n-1},$$

*the marginal density of the r-th record value.*

*For progressive type II censored order statistics with equi-balanced censoring scheme, the form of the marginal density is the same as the form of (3), with m=R, R being the removal number.*

### 2.2. Concomitants

The term concomitant was introduced by David (1973) [1] and has the following definition:

**Definition** **2.**  *Let $\left(X_{1},Y_{1}\right),\left(X_{2},Y_{2}\right),\ldots ,\left(X_{n},Y_{n}\right)$ be iid bivariate random variables with cumulative distribution function F(x,y). Then, the Y variate associated to the r-th-order statistic of X-s, $X_{\left(r:n\right)}$, denoted by $Y_{\left[r:n\right]}$, is the concomitant of $X_{\left(r:n\right)}$.*

A natural use of concomitants is in selection procedures when *k* individuals are chosen on the basis of their *X*-values. Then, the corresponding *Y*-values represent performance on an associated characteristics. In Reliability Theory, the role of the concomitants is emphasized in [3,4,5].

### 2.3. Concomitants of FGM Family

The FGM bivariate distribution family has a flexible form and it was studied by Farlie [16], Gumbel [17], Morgenstern [18], and Johnson and Kotz [19].

**Definition** **3.**  *Let X and Y be two random variables with distribution functions $F_{X}$ and $F_{Y}$, respectively. Additionally, let α be a real number. Then, the FGM family has the distribution function:*

(5) $$F_{X,Y}\left(x,y\right)=F_{X}\left(x\right)F_{Y}\left(y\right)\left[1+\alpha \left(1-F_{X}\left(x\right)\right)\left(1-F_{Y}\left(y\right)\right)\right].$$

The corresponding probability density function (pdf) of (5) is:

(6) $$f_{X,Y}\left(x,y\right)=f_{X}\left(x\right)f_{Y}\left(y\right)\left[1+\alpha \left(1-F_{X}\left(x\right)\right)\left(1-F_{Y}\left(y\right)\right)\right],$$

where $f_{X}\left(x\right)$$f_{Y}\left(y\right)$ are the marginals of $f_{X,Y}\left(x,y\right)$.

The parameter α∈[−1,1] is known as the association parameter and the two random variables *X* and *Y* are independent when α=0. For α≠0, there is a dependence between the two variables, characterized by the FGM-copula whose properties were studied in [20].

Concomitants of FGM family, related to GOS, started to come into notice with the work of Beg and Ahsanullah in 2008 [21] where the density $g_{\left[r,n,m,k\right]}$ of the concomitant of the r-th GOS is derived:

(7) $$g_{\left[r,n,m,k\right]}\left(y\right)=f_{Y}\left(y\right)+\alpha \left(2F_{Y}\left(y\right)-1\right)f_{Y}\left(y\right)C^{*}\left(r,n,m,k\right),$$

where

$$C^{*}\left(r,n,m,k\right)=1-2\frac{c_{r-1}}{\left(\gamma_{1}+1\right)\left(\gamma_{2}+1\right)\ldots \left(\gamma_{r}+1\right)}$$

is a constant.

**Remark** **2.**  If m=0, k=1, then $C^{*}\left(r,n,0,1\right)=-\left(n-2r+1\right)/\left(n+1\right)$ and

(8) $$g_{\left[r,n,0,1\right]}\left(y\right)=f_{Y}\left(y\right)-\alpha \frac{n-2r+1}{n+1}\left(2F_{Y}\left(y\right)-1\right)f_{Y}\left(y\right)$$

*is the density of the concomitant of r-th-order statistic from the FGM family.*

If m=−1, k=1, then $C^{*}\left(r,n,-1,1\right)=1-2^{1-r}$ and

(9) $$g_{\left[r,n,-1,1\right]}\left(y\right)=f_{Y}\left(y\right)-\alpha \left(2^{1-r}-1\right)\left(2F_{Y}\left(y\right)-1\right)f_{Y}\left(y\right).$$

*If we are in the case of progressive type II censoring order statistics with equi-balanced censoring scheme, the density of the concomitant of r-th-order statistic from the FGM family is (7) with m=R, the removal number.*

The cumulative distribution function and the survival function of the concomitant of r-th-order statistic can also be computed:

(10) $$\begin{array}{cc} G_{\left[r,n,m,k\right]}\left(y\right) & =f_{Y}\left(y\right)+\alpha \left(1-F_{Y}\left(y\right)\right)f_{Y}\left(y\right)C^{*}\left(r,n,m,k\right), \end{array}$$

(11) $$\begin{array}{cc} {\bar{G}}_{\left[r,n,m,k\right]}\left(y\right) & =1-f_{Y}\left(y\right)-\alpha \left(1-F_{Y}\left(y\right)\right)f_{Y}\left(y\right)C^{*}\left(r,n,m,k\right). \end{array}$$

In the following, in order to make it easier to read computations, we make the notations: $Y_{\left[r\right]}^{*}=Y_{\left[r,n,m,k\right]}$, $g_{\left[r\right]}=g_{\left[r,n,m,k\right]}$, $G_{\left[r\right]}=G_{\left[r,n,m,k\right]}$, ${\bar{G}}_{\left[r\right]}={\bar{G}}_{\left[r,n,m,k\right]}$, $C_{r}^{*}=C^{*}\left(r,n,m,k\right)$.

## 3. Information Measures for the Concomitants from the FGM Family, Existing Results

In this section, we will recall some definitions and results for the information measures of the concomitants of GOS from the FGM family.

### 3.1. Shannon and Shannon-Related Entropies

**Shannon entropy** was introduced by Shannon in 1948 [22], it has multiple applications and it can be defined as:

(12) $$H^{S}\left(X\right)=-E\left[\log f\left(X\right)\right]$$

Information measures for concomitants derived from the FGM family have been studied by Tahmasebi and Behboodian: in [23] for concomitants of order statistics, and in [24] for concomitants of GOS. Using (7), they proved that for the Shannon entropy of $Y_{\left[r\right]}$, the concomitant of the r-th generalized order statistics is:

(13) $$H^{S}\left(Y_{\left[r\right]}^{*}\right)=W\left(r,\alpha ,n,m,k\right)+H^{S}\left(Y\right)\left(1-\alpha C_{r}^{*}\right)-2\alpha C_{r}^{*}\phi_{f}\left(u\right),$$

where

(14) $$\begin{array}{cc} W(r,\alpha ,n,m,k)= & \frac{1}{4\alpha C_{r}^{*}}\left\{{\left(1-C_{r}^{*}\alpha \right)}^{2}\log\left(1-C_{r}^{*}\alpha \right)-{\left(1+C_{r}^{*}\alpha \right)}^{2}\log\left(1+C_{r}^{*}\alpha \right)\right\}+\frac{1}{2}, \end{array}$$

and

(15) $$\phi_{f}\left(u\right)=\int_{0}^{1}\log f_{Y}\left(F_{Y}^{-1}\left(u\right)\right)du.$$

**Remark** **3.**  *In [23] are analyzed the properties of (13) in the particular case when m=0 and k=1, i.e., when the GOS reduces to the simple order statistics, and therefore, the entropy from (13) in this case is the Shannon entropy of the concomitant of r-th-order statistic:*

(16) $$H^{S}\left(Y_{\left[r\right]}\right)=W\left(r,\alpha ,n,0,1\right)+H^{S}\left(Y\right)\left(1+\alpha \frac{n-2r+1}{n+1}\right)+2\alpha \frac{n-2r+1}{n+1}\phi_{f}\left(u\right)$$

*In [24], Shannon entropy for record values is also mentioned:*

(17) $$H^{S}\left(R_{\left[r\right]}\right)=W\left(r,\alpha ,n,-1,1\right)+H^{S}\left(Y\right)\left(1+\alpha \left(2^{1-r}-1\right)\right)+2\alpha \left(2^{1-r}-1\right)\phi_{f}\left(u\right).$$

*If we are in the case of progressive type II censoring order statistics with an equi-balanced censoring scheme, the Shannon entropy of the concomitant of r-th-order statistic from the FGM family is (13) with m=R, the removal number.*

Awad, in 1987, ref. [25] noticed that Shannon entropy, in the continuous case, does not fulfill the condition that the entropy is preserved under the linear transformation and proposed the following entropy known also in the literature as Sup-entropy:

(18) $$H^{SA}\left(X\right)=-E\left[\log\frac{f(X)}{\delta }\right]$$

where δ=sup{f(x)|x∈R}. We will call this entropy **Shannon–Awad entropy**.

**Residual and past Shannon entropies** were defined in the context of reliability, being important in measuring the amount of information that a residual life or a past life of a unit has. In the following, the random variable *X* with pdf *f*, cdf *F*, and survival function $\bar{F}$, is considered positive and it has the meaning of a lifetime of a unit.

Residual entropy is introduced and its properties are analyzed in the works of Ebrahimi [26] and Ebrahimi and Pellerey [27]. Residual entropy is based on the idea of measuring the expected uncertainty contained in the conditional density of X−t given X>t [27]:

(19) $$H^{S}\left(X;t\right)=-E\left[\log\frac{f(X)}{\bar{F}\left(t\right)}|X>t\right].$$

In terms of failure rate, the residual entropy can be written as:

$$H^{S}\left(X;t\right)=1-E\left[\log\lambda_{F}\left(X\right)|X>t\right],$$

where $\lambda_{F}\left(\cdot \right)=f\left(\cdot \right)/\bar{F}\left(\cdot \right)$ is the failure rate function.

Similar to the definition of the residual entropy, DiCrescenzo and Longobardi [28] introduced past entropy as a dual to the residual entropy. Past entropy measures the uncertainty about past life of a failed unit:

(20) $${\bar{H}}^{S}\left(X;t\right)=-E\left[\log\frac{f(X)}{F(t)}|X<t\right].$$

In terms of reversed failure rate, past entropy can be written as:

$${\bar{H}}^{S}\left(X;t\right)=1-E\left[\log\tau_{F}\left(X\right)|X<t\right],$$

where $\tau_{F}\left(\cdot \right)=f\left(\cdot \right)/F\left(\cdot \right)$ is the reversed failure rate function.

Residual and past entropies for concomitants of GOS from the FGM family were determined by Mohie EL-Din et al. in [29]. They considered also concomitants of other types of GOS, but the form of the entropies is similar. The residual entropy of the r-th concomitant of GOS from the FGM family is [29]:

(21) $$\begin{array}{cc} H^{S}\left(Y_{\left[r\right]}^{*};t\right)=\log{\bar{G}}_{\left[r\right]}\left(t\right)-\frac{1}{{\bar{G}}_{\left[r\right]}\left(t\right)}\{ & \left(1-\alpha C_{r}^{*}\right)\left[{\bar{F}}_{Y}\left(t\right)\left(\log{\bar{F}}_{Y}\left(t\right)-H^{S}\left(Y;t\right)\right)\right]+\\ \; & +2\alpha C_{r}^{*}\phi_{f}\left(t\right)+K_{1}\left(r,t,\alpha ,n,m,k\right)\}, \end{array}$$

where

(22) $$\begin{array}{cc} K_{1}(r,t, & \alpha ,n,m,k)=\frac{1}{2\alpha C_{r}^{*}}\{\frac{-1}{4}\left[{\left(1+\alpha C_{r}^{*}\right)}^{2}-{\left(1+\alpha C_{r}^{*}\left(2F_{Y}\left(t\right)-1\right)\right)}^{2}\right]+\\ \; & +\frac{1}{2}\left[{\left(1+\alpha C_{r}^{*}\right)}^{2}\log\left(1+\alpha C_{r}^{*}\right)-{\left(1+\alpha C_{r}^{*}\left(2F_{Y}\left(t\right)-1\right)\right)}^{2}\log\left(1+\alpha C_{r}^{*}\left(2F_{Y}\left(t\right)-1\right)\right)\right]\}, \end{array}$$

and

(23) $$\phi_{f}\left(t\right)=\int_{t}^{\infty }F_{Y}\left(y\right)f_{Y}\left(y\right)\log f_{Y}\left(y\right)dy.$$

We notice that for t=0, the residual entropy (21) becomes the entropy (13).

**Remark** **4.**  *For m=0 and k=1, we obtain residual Shannon entropy for the concomitant of r-th-order statistic:*

(24) $$\begin{array}{cc} H^{S}\left(Y_{\left[r\right]};t\right)=\log{\bar{G}}_{\left[r\right]}\left(t\right)-\frac{1}{{\bar{G}}_{\left[r\right]}\left(t\right)}\{ & \left(1-\alpha \frac{n-2r+1}{n+1}\right)\left[{\bar{F}}_{Y}\left(t\right)\left(\log{\bar{F}}_{Y}\left(t\right)-H^{S}\left(Y;t\right)\right)\right]-\\ \; & -2\alpha \frac{n-2r+1}{n+1}\phi_{f}\left(t\right)+K_{1}\left(r,t,\alpha ,n,0,1\right)\}. \end{array}$$

*For m=−1 and k=1, we obtain residual Shannon entropy for the concomitant of r-th record value:*

(25) $$\begin{array}{cc} H^{S}\left(R_{\left[r\right]};t\right)=\log{\bar{G}}_{\left[r\right]}\left(t\right)-\frac{1}{{\bar{G}}_{\left[r\right]}\left(t\right)}\{ & \left(1-\alpha \left(2^{1-r}-1\right)\right)\left[{\bar{F}}_{Y}\left(t\right)\left(\log{\bar{F}}_{Y}\left(t\right)-H^{S}\left(Y;t\right)\right)\right]-\\ \; & -2\alpha \left(2^{1-r}-1\right)\phi_{f}\left(t\right)+K_{1}\left(r,t,\alpha ,n,-1,1\right)\}. \end{array}$$

*In the case of progressive type II censoring order statistics with equi-balanced censoring scheme, the residual Shannon entropy of the concomitant of r-th-order statistic from the FGM family is (21) with m=R, the removal number.*

In a similar way, the past entropy for the concomitant of the r-th GOS from the FGM family is defined as [29]:

(26) $$\begin{array}{cc} {\bar{H}}^{S}\left(Y_{\left[r\right]}^{*};t\right)=\log G_{\left[r\right]}\left(t\right)-\frac{1}{G_{\left[r\right]}\left(t\right)}\{ & \left(1-\alpha C_{r}^{*}\right)\left[F_{Y}\left(t\right)\left(\log F_{Y}\left(t\right)-{\bar{H}}^{S}\left(Y;t\right)\right)\right]+\\ \; & +2\alpha C_{r}^{*}{\bar{\phi }}_{f}\left(t\right)+K_{2}\left(r,t,\alpha ,n,m,k\right)\}, \end{array}$$

where

(27) $$\begin{array}{cc} K_{2}(r,t, & \alpha ,n,m,k)=\frac{1}{2\alpha C_{r}^{*}}\{\frac{-1}{4}\left[{\left(1+\alpha C_{r}^{*}\left(2F_{Y}\left(t\right)-1\right)\right)}^{2}-{\left(1-\alpha C_{r}^{*}\right)}^{2}\right]+\\ \; & +\frac{1}{2}\left[{\left(1+\alpha C_{r}^{*}\left(2F_{Y}\left(t\right)-1\right)\right)}^{2}\log\left(1+\alpha C_{r}^{*}\left(2F_{Y}\left(t\right)-1\right)\right)-{\left(1-\alpha C_{r}^{*}\right)}^{2}\log\left(1-\alpha C_{r}^{*}\right)\right]\}, \end{array}$$

and

(28) $${\bar{\phi }}_{f}\left(t\right)=\int_{0}^{t}F_{Y}\left(y\right)f_{Y}\left(y\right)\log f_{Y}\left(y\right)dy.$$

We notice that for t→∞, the past entropy (26) becomes the entropy (13).

### 3.2. Tsallis and Tsallis-Related Entropies

For the first time, Tsallis entropy was introduced and used in the context of Cybernetics Theory by Harvda and Charvat [30], but it has become well known since its definition as a generalization of Boltzmann–Gibbs statistics, in the context of thermodynamics, by Tsallis in 1988 [31]. Being the starting point of the field of non-extensive statistics, Tsallis entropy is a non-additive generalization of the Shannon entropy and for a continuous random variable *X* with density function *f*, it can be defined as:

(29) $$H^{T}\left(X\right)=\frac{1}{q-1}\left\{1-\int_{-\infty }^{+\infty }{\left[f\left(x\right)\right]}^{q}dx\right\}\;\;q>0,\;q\neq 1.$$

When q→1, Tsallis entropy approaches to Shannon entropy. Tsallis entropy has, in turn, various generalizations, see, for example, [32].

Another important element in non-extensive statistics is $\log_{q}$ function:

(30) $$\log_{q}x=\frac{x^{1-q}-1}{1-q},\;\;x>0,\;q\neq 1,$$

and Tsallis entropy can be obtained using this function in two ways:

$$H^{T}\left(X\right)=E\left[\log_{q}\frac{1}{f(X)}\right]=\frac{1}{q-1}E\left[1-{\left[f\left(X\right)\right]}^{q-1}\right].$$

Tsallis entropy has applications in many fields, from statistical mechanics and thermodynamics, to image processing and reliability, sometimes being more suited to measuring uncertainty than classical Shannon entropy [33,34].

In [35], Tsallis entropy was computed and its properties obtained for record values and their concomitants when the bivariate distribution is in the FGM family.

Similar to the Shannon case, we can think about residual and past variants of the Tsallis entropy in the context of reliability. In [36], Nanda and Paul introduced **residual Tsallis entropy** as the ’first kind residual entropy of order β’. In our notation, β is *q*:

(31) $$H^{T}\left(X;t\right)=\frac{1}{q-1}\left\{1-\int_{t}^{\infty }{\left[\frac{f(x)}{\bar{F}\left(t\right)}\right]}^{q}dx\right\}=\frac{1}{q-1}\left\{1-\frac{1}{{\left[\bar{F}\left(t\right)\right]}^{q}}\int_{t}^{\infty }{\left[f\left(x\right)\right]}^{q}dx\right\}.$$

In addition to entropy type information measures, there are another two types information measures that can be associated to probability distributions—Fisher measures and divergence measures [37].

### 3.3. Fisher Information Number

Fisher information measures the amount of information that we can obtain from a sample about an unknown parameter and therefore, it measures the uncertainty included in a unknown characteristic of a population. If the parameter is a location one, then Fisher information is shift-invariant and has the form:

(32) $$I_{f}=\int_{-\infty }^{+\infty }{\left(\frac{\partial }{\partial x}\log f\left(x\right)\right)}^{2}f\left(x\right)dx=E\left[{\left(\frac{\partial }{\partial x}\log f\left(x\right)\right)}^{2}\right].$$

Shift-invariant Fisher information, also called Fisher Information Number (FIN), was studied in [38]. It has applications in statistical physics where it is also known by the name extreme physical information [39], and it is used in analyzing the evolution of dynamical systems.

For the concomitants of GOS from the FGM family, the Fisher information number was determined in [40].

### 3.4. Divergence Measures

Divergences are useful tools when a measure of the difference between two probability distributions is needed and therefore, they have applications in various fields, from inference for Markov chains, [41,42,43] to machine learning [44,45]. One of the best-known divergence is Kullback–Leibler divergence [46,47], which for two continuous random variables $Z_{1}$, with probability density $f_{1}$, and $Z_{2}$ with probability density $f_{2}$ is:

(33) $$KLD\left(Z_{1},Z_{2}\right)=\int_{-\infty }^{+\infty }f_{1}\left(z\right)log\left(\frac{f_{1}\left(z\right)}{f_{2}\left(z\right)}\right)dz.$$

Kullback–Leibler divergence for the concomitants of GOS from the FGM family was computed in [24] and the result is distribution-free.

One of the generalizations of Kullback–Leibler divergence measure is Tsallis divergence, which expands Kullback–Leibler divergence in a similar way to that in which Tsallis entropy extends Shannon entropy. There is a very rich literature on Tsallis divergence or Tsallis relative entropy in the case of the discrete distributions, see, for example, [48,49,50]. Tsallis divergence for continuous distributions does not appear so frequently in the literature, being studied mainly in Machine Learning context, [44,45]. Tsallis divergence for the concomitants will be determined in this paper in the next section.

## 4. Information Measures for the Concomitants from FGM Family, New Results

In this section, we will provide some generalizations of the existing results on the information measures for the concomitants of GOS from the FGM family, results that are mentioned in previous section. We are interested in Awad-type extensions of the entropies, in residual and past Tsallis entropies, in Tsallis type extension of the FIN, and in Tsallis divergence.

### 4.1. Shannon and Shannon-Related Entropies

One can easily notice that the relationship between Shannon–Awad entropy (18) and Shannon entropy (12) is:

(34) $$H^{SA}\left(X\right)=H^{S}\left(X\right)+\log\delta .$$

In the following, we provide natural extensions of the results obtained in [24], considering Shannon–Awad entropy instead of Shannon entropy. Thus, **Shannon–Awad entropy** of the concomitant of the r-th GOS from the FGM family is:

(35) $$\begin{array}{cc} H^{SA}\left(Y_{\left[r\right]}^{*}\right)= & W\left(r,\alpha ,n,m,k\right)+\left(H^{SA}\left(Y\right)-\log\delta \right)\left(1-\alpha C_{r}^{*}\right)-2\alpha C_{r}^{*}\phi_{f}\left(u\right)+\log\delta^{\left[r\right]}, \end{array}$$

where W(r,α,n,m,k) and $\phi_{f}$ are given by (14) and (15) and

$$\delta =\sup\left\{f_{Y}\left(x\right)|x>0\right\},\;\;\;\delta^{\left[r\right]}=\sup\left\{g_{\left[r\right]}\left(x\right)|x>0\right\}.$$

**Remark** **5.**  *For the simple OS, the r-th OS concomitant from the FGM family, Shannon–Awad entropy is:*

(36) $$\begin{array}{cc} H^{SA}\left(Y_{\left[r\right]}\right) & =W\left(r,\alpha ,n,0,1\right)+\left(H^{SA}\left(Y\right)-\log\delta \right)\left(1+\alpha \frac{n-2r+1}{n+1}\right)+\\ \; & +2\alpha \frac{n-2r+1}{n+1}\phi_{f}\left(u\right)+\log\delta_{\left[r\right]}, \end{array}$$

*with $\delta_{\left[r\right]}=\sup\left\{g_{\left[r\right]}\left(x\right)|x>0\right\}$ and $g_{\left[r\right]}$ being here the pdf of the concomitant r-th-order statistics.*

*For the record values, the r-th record value concomitant from the FGM family, Shannon–Awad entropy is:*

(37) $$\begin{array}{cc} H^{SA}\left(R_{\left[r\right]}\right) & =W\left(r,\alpha ,n,-1,1\right)+\left(H^{SA}\left(Y\right)-\log\delta \right)\left[1+\alpha (2^{1-r}-1)\right]+\\ \; & +2\alpha \left(2^{1-r}-1\right)\phi_{f}\left(u\right)+\log\delta_{\left[r\right]} \end{array}$$

*with $\delta_{\left[r\right]}=\sup\left\{g_{\left[r\right]}\left(x\right)|x>0\right\}$ and $g_{\left[r\right]}$ being here the pdf of the concomitant r-th record value.*

*In the case of progressive type II censoring order statistics with equi-balanced censoring scheme, the Shannon–Awad entropy of the concomitant of r-th-order statistic from the FGM family is (35) with m=R, the removal number.*

An extension of the above entropies are **residual and past Shannon–Awad entropies**. We define residual Shannon–Awad entropy as:

(38) $$H^{SA}\left(X;t\right)=-E\left[\log\frac{f(X)}{\bar{F}\left(t\right)\delta_{\left(t,\infty \right)}^{\bar{F}}}|X>t\right],$$

where

(39) $$\delta_{\left(t,\infty \right)}^{\bar{F}}=\frac{1}{\bar{F}\left(t\right)}\sup\left\{f\left(x\right)|x\in \left(t,\infty \right)\right\}.$$

In terms of failure rate, residual Shannon–Awad entropy can be obtained:

(40) $$H^{SA}\left(X;t\right)=1-E\left[\log\lambda \left(X\right)|X>t\right]+\log\delta_{\left(t,\infty \right)}^{\bar{F}}.$$

We notice that the relationship between residual Shannon entropy and residual Shannon–Awad entropy is similar to (34) and it is:

(41) $$H^{SA}\left(X;t\right)=H^{S}\left(X;t\right)+\log\delta_{\left(t,\infty \right)}^{\bar{F}}.$$

In a similar way, we can extend past Shannon entropy to past Shannon–Awad entropy:

(42) $${\bar{H}}^{SA}\left(X;t\right)=-E\left[\log\frac{f(X)}{F\left(t\right)\delta_{\left(0,t\right)}^{F}}|X<t\right],$$

where

(43) $$\delta_{\left(0,t\right)}^{F}=\frac{1}{F(t)}\sup\left\{f\left(x\right)|x\in \left(0,t\right)\right\}.$$

As a function of reversed failure rate, past Shannon–Awad entropy can be written:

(44) $${\bar{H}}^{SA}\left(X;t\right)=1-E\left[\log\tau \left(X\right)|X<t\right]+\log\delta_{\left(0,t\right)}^{F}.$$

We can write also the relationship between past Shannon–Awad entropy and past Shannon entropy:

(45) $${\bar{H}}^{SA}\left(X;t\right)={\bar{H}}^{S}\left(X;t\right)+\log\delta_{\left(0,t\right)}^{F}.$$

Taking into account the above relationships, (21), and (26), we can obtain the Awad-type extension of the Shannon entropy for the concomitant of r-th GOS from the FGM family, when the concomitant represents the residual life or the past life of a unit.

**Theorem** **1.**  *Residual Shannon–Awad entropy for the concomitant of r-th GOS from the FGM family is:*

(46) $$\begin{array}{cc} H^{SA}\left(Y_{\left[r\right]}^{*};t\right)=\log{\bar{G}}_{\left[r\right]}\left(t\right)-\frac{1}{{\bar{G}}_{\left[r\right]}\left(t\right)}\{ & \left(1-\alpha C_{r}^{*}\right)\left[{\bar{F}}_{Y}\left(t\right)\left(\log{\bar{F}}_{Y}\left(t\right)-H^{SA}\left(Y;t\right)+\delta_{t,\infty }^{{\bar{F}}_{Y}}\right)\right]+\\ \; & +2\alpha C_{r}^{*}\phi_{f}\left(t\right)+K_{1}\left(r,t,\alpha ,n,m,k\right)\}+\log\delta_{\left(t,\infty \right)}^{{\bar{G}}_{\left[r\right]}} \end{array}$$

*where $K_{1}\left(r,t,\alpha ,n,m,k\right)$ and $\phi_{f}\left(t\right)$ are given by (22), (23) respectively, and*

$$\delta_{\left(t,\infty \right)}^{{\bar{F}}_{Y}}=\frac{1}{{\bar{F}}_{Y}\left(t\right)}\sup\left\{f_{Y}\left(x\right)|x\in \left(t,\infty \right)\right\},\;\;\;\delta_{\left(t,\infty \right)}^{{\bar{G}}_{\left[r\right]}}=\frac{1}{{\bar{G}}_{\left[r\right]}\left(t\right)}\sup\left\{g_{\left[r\right]}\left(x\right)|x\in \left(t,\infty \right)\right\}.$$

*Past Shannon–Awad entropy for the concomitant of r-th GOS from the FGM family is:*

(47) $$\begin{array}{cc} {\bar{H}}^{SA}\left(Y_{\left[r\right]}^{*};t\right)=\log G_{\left[r\right]}\left(t\right)-\frac{1}{G_{\left[r\right]}\left(t\right)}\{ & \left(1-\alpha C_{r}^{*}\right)\left[F_{Y}\left(t\right)\left(\log F_{Y}\left(t\right)-{\bar{H}}^{SA}\left(Y;t\right)+\delta_{\left(0,t\right)}^{F_{Y}}\right)\right]+\\ \; & +2\alpha C_{r}^{*}{\bar{\phi }}_{f}\left(t\right)+K_{2}\left(r,t,\alpha ,n,m,k\right)\}+\log\delta_{\left(0,t\right)}^{G_{\left[r\right]}} \end{array}$$

*where $K_{2}\left(r,t,\alpha ,n,m,k\right)$ and ${\bar{\phi }}_{f}\left(y\right)$, and $\delta_{\left(0,t\right)}$ are given by (22) and (23) respectively, and*

$$\delta_{\left(0,t\right)}^{F_{Y}}=\frac{1}{F_{Y}\left(t\right)}\sup\left\{f_{Y}\left(x\right)|x\in \left(0,t\right)\right\},\;\;\;\delta_{\left(0,t\right)}^{G_{\left[r\right]}}=\frac{1}{G_{\left[r\right]}\left(t\right)}\sup\left\{g_{\left[r\right]}\left(x\right)|x\in \left(0,t\right)\right\}.$$

**Corollary** **1.**  *The residual Shannon–Awad entropy for the concomitant of r-th-order statistic is:*

(48) $$\begin{array}{cc} H^{SA}\left(Y_{\left[r\right]};t\right)=\log{\bar{G}}_{\left[r\right]}\left(t\right)-\frac{1}{{\bar{G}}_{\left[r\right]}\left(t\right)}\{ & \left(1+\alpha \frac{n-2r+1}{n+1}\right)\left[{\bar{F}}_{Y}\left(t\right)\left(\log{\bar{F}}_{Y}\left(t\right)-H^{SA}\left(Y;t\right)+\delta_{t,\infty }^{{\bar{F}}_{Y}}\right)\right]+\\ \; & -2\alpha \frac{n-2r+1}{n+1}\phi_{f}\left(t\right)+K_{1}\left(r,t,\alpha ,n,0,1\right)\}+\log\delta_{\left(t,\infty \right)}^{{\bar{G}}_{\left[r\right]}}. \end{array}$$

*The residual Shannon–Awad entropy for the concomitant of r-th record value is:*

(49) $$\begin{array}{cc} H^{SA}\left(R_{\left[r\right]};t\right)=\log{\bar{G}}_{\left[r\right]}\left(t\right)-\frac{1}{{\bar{G}}_{\left[r\right]}\left(t\right)}\{ & \left(1+\alpha \left(2^{1-r}-1\right)\right)\left[{\bar{F}}_{Y}\left(t\right)\left(\log{\bar{F}}_{Y}\left(t\right)-H^{SA}\left(Y;t\right)+\delta_{t,\infty }^{{\bar{F}}_{Y}}\right)\right]+\\ \; & -2\alpha \left(2^{1-r}-1\right)\phi_{f}\left(t\right)+K_{1}\left(r,t,\alpha ,n,-1,1\right)\}+\log\delta_{\left(t,\infty \right)}^{{\bar{G}}_{\left[r\right]}}. \end{array}$$

*In the case of progressive type II censoring order statistics with equi-balanced censoring scheme, the residual Shannon–Awad entropy of the concomitant of r-th-order statistic from the FGM family is (46) with m=R, the removal number.*

*Similar results can be obtained also for past Shannon–Awad entropy.*

### 4.2. Tsallis and Tsallis-Related Entropies

Information measures related to Tsallis entropy for the concomitants are very few in the literature. In [35], Tsallis entropy and residual Tsallis entropy for the concomitants of the record values from the FGM family are obtained. In this subsection, we will obtain more general results, computing Tsallis entropies for the concomitants of generalized order statistics and, furthermore, considering Awad-type extensions of the Tsallis entropies.

**Theorem** **2.**  *Tsallis entropy for the concomitant of the r-th GOS from the FGM family is:*

(50) HT(Y[r]*)=1q−11−∑k=0q∑s=0k(−1)sqkks2k−sαkCr*kEUfY(FY−1(U))q−1Uk−s,

*where U is an U(0,1) random variable and $E_{U}$ is the expectation of $f_{Y}{\left(F_{Y}^{-1}\left(U\right)\right)}^{q-1}U^{k-s}$.*

**Proof.**  Taking into account the definitions of Tsallis entropy (29) and the density of the concomitants (6), we obtain:

$$\begin{array}{cc} H^{T}\left(Y_{\left[r\right]}^{*}\right) & =\frac{1}{q-1}\left\{1-\int_{0}^{\infty }{\left[g_{\left[r\right]}\left(y\right)\right]}^{q}dy\right\}=\\ \; & =\frac{1}{q-1}\left(1-\int_{0}^{\infty }{\left[f_{Y}\left(y\right)\right]}^{q}{\left(1+\alpha C_{r}^{*}\left(2F_{Y}\left(y\right)-1\right)\right)}^{q}dy\right). \end{array}$$

We have that:

(1+αCr*(2FY(y)−1))q=∑k=0q∑s=0k(−1)sqkks2k−sαkCr*k[FY(y)]k−s.

Additionally, if we consider the transformation

$$F_{Y}\left(y\right)=u;\;\;y=F_{Y}^{-1}\left(u\right),\;\;f_{Y}\left(y\right)dy=du,$$

the result (50) follows. □

**Corollary** **2.**  *The Tsallis entropy for the concomitant of r-th-order statistic is:*

(51) HT(Y[r])=1q−1{1−∑k=0q∑s=0k(−1)sqkks2k−sαk(−1)kn−2r+1n+1k××EUfY(FY−1(U))q−1Uk−s}.

*The Tsallis entropy for the concomitant of r-th record value is:*

(52) HT(R[r])=1q−1{1−∑k=0q∑s=0k(−1)sqkks2k−sαk(−1)k21−r−1k××EUfY(FY−1(U))q−1Uk−s}.

*In the case of progressive type II censoring order statistics with equi-balanced censoring scheme, the Tsallis entropy of the concomitant of r-th-order statistic from the FGM family is (50) with m=R, the removal number.*

We now discuss some Tsallis-related entropies. First, we give an Awad-type extension of Tsallis entropy and then we focus on Residual Tsallis, Past Tsallis entropies and their Awad-type extensions.

Several Awad-type extensions have been proposed in the literature ([51,52]). Now, we introduce this type of extension for Tsallis entropy and we define **Tsallis–Awad entropy** for a continuous random variable *X* which take values in R:

(53) $$H^{TA}\left(X\right)=\frac{1}{q-1}\left\{1-\int_{-\infty }^{+\infty }{\left[\frac{f(x)}{\delta }\right]}^{q-1}f\left(x\right)dx\right\}=\frac{1}{q-1}\left\{1-\frac{1}{\delta^{q-1}}\int_{-\infty }^{+\infty }{\left[f\left(x\right)\right]}^{q}dx\right\},$$

where δ=sup{f(x)|x∈R}.

We notice that the relationship between Tsallis–Awad entropy and Tsallis entropy is:

(54) $$H^{TA}\left(X\right)=\delta^{1-q}H^{T}\left(X\right)+\log_{q}\delta .$$

Using (50) and (54), we can obtain the expression of Tsallis–Awad entropy for the concomitant of the r-th GOS from the FGM family:

**Theorem** **3.**  *Tsallis–Awad entropy for the concomitant of the r-th GOS from the FGM family is:*

(55) HTA(Y[r]*)=δ1−qq−11−∑k=0q∑s=0k(−1)sqkks2k−sαkCr*kEUfY(FY−1(U))q−1Uk−s+logqδ,

*where U is an U(0,1) random variable, and, in this case, $\delta =\sup\{g_{\left[r\right]}\left(x\right)|x>0\}$.*

**Corollary** **3.**  *The Tsallis–Awad entropy for the concomitant of r-th-order statistic is:*

(56) HT(Y[r])=δ1−qq−1{1−∑k=0q∑s=0k(−1)sqkks2k−sαk(−1)kn−2r+1n+1k××EUfY(FY−1(U))q−1Uk−s}+logqδ.

*The Tsallis–Awad entropy for the concomitant of r-th record value is:*

(57) HT(R[r])=δ1−qq−1{1−∑k=0q∑s=0k(−1)sqkks2k−sαk(−1)k21−r−1k××EUfY(FY−1(U))q−1Uk−s}+logqδ.

*In the case of progressve type II censoring order statistics with equi-balanced censoring scheme, the Tsallis–Awad entropy of the concomitant of r-th-order statistic from the FGM family is (55) with m=R, the removal number.*

In a similar way to the definition of **residual Tsallis** (31), we can consider the **past Tsallis entropy**:

(58) $${\bar{H}}^{T}\left(X;t\right)=\frac{1}{q-1}\left\{1-\int_{0}^{t}{\left[\frac{f(x)}{F(t)}\right]}^{q}dx\right\}=\frac{1}{q-1}\left\{1-\frac{1}{F{\left(t\right)}^{q}}\int_{0}^{t}{\left[f\left(x\right)\right]}^{q}dx\right\}.$$

Taking into account Theorem 2, the following theorem is naturally deduced:

**Theorem** **4.**  *Residual Tsallis entropy for the concomitant of the r-th GOS from the FGM family is:*

(59) HT(Y[r]*;t)=1q−1{1−1G¯[r](t)q∑k=0q∑s=0k(−1)sqkks2k−sαkCr*k××EUfY(F−1(U))q−1Uk−s|U>FY(t)},

*where U is an U(0,1) random variable and $E_{U}$ is the conditional expectation of $f_{Y}{\left(F^{-1}\left(U\right)\right)}^{q-1}U^{k-s}$, given $U>F_{Y}\left(t\right)$.*

*Past Tsallis entropy for the concomitant of the r-th GOS from the FGM family is:*

(60) H¯T(Y[r]*;t)=1q−1{1−1G[r](t)q∑k=0q∑s=0k(−1)sqkks2k−sαkCr*k××EUfY(FY−1(U))q−1Uk−s|U<FY(t)},

*where U is a U(0,1) random variable and $E_{U}$ is the conditional expectation of $f_{Y}{\left(F^{-1}\left(U\right)\right)}^{q-1}U^{k-s}$, given $U<F_{Y}\left(t\right)$.*

**Corollary** **4.**  *The residual Tsallis entropy for the concomitant of r-th-order statistic is:*

(61) HT(Y[r];t)=1q−1{1−1G¯[r](t)q∑k=0q∑s=0k(−1)sqkks2k−sαk(−1)kn−2r+1n+1k××EUfY(F−1(U))q−1Uk−s|U>FY(t)},

*The residual Tsallis entropy for the concomitant of r-th record value is:*

(62) HT(R[r];t)=1q−1{1−1G¯[r](t)q∑k=0q∑s=0k(−1)sqkks2k−sαk(−1)k21−r−1k××EUfY(F−1(U))q−1Uk−s|U>FY(t)}.

*In the case of progressive type II censoring order statistics with equi-balanced censoring scheme, the residual Tsallis entropy of the concomitant of r-th-order statistic from the FGM family is (59) with m=R, the removal number.*

*Similar results can be obtained for past Tsallis entropy.*

We now introduce **Residual Tsallis–Awad entropy**:

(63) $$\begin{array}{cc} H^{TA}\left(X;t\right) & =\frac{1}{q-1}\left\{1-\frac{1}{{\left(\delta_{\left(t,+\infty \right)}^{\bar{F}}\right)}^{q-1}}\int_{t}^{+\infty }{\left[\frac{f(x)}{\bar{F}\left(t\right)}\right]}^{q}dx\right\} \end{array}$$

(64) $$\begin{array}{cc} \; & =\frac{1}{q-1}\left\{1-\frac{1}{\bar{F}\left(t\right)}E\left[{\left[\frac{f(X)}{\bar{F}\left(t\right)\delta_{\left(t,\infty \right)}^{\bar{F}}}\right]}^{q-1}|X>t\right]\right\}, \end{array}$$

where $\delta_{\left(t,\infty \right)}^{\bar{F}}=\frac{1}{\bar{F}\left(t\right)}\sup\left\{f\left(x\right)|x\in \left(t,+\infty \right)\right\}$.

**Past Tsallis–Awad entropy** can also be defined:

(65) $$\begin{array}{cc} {\bar{H}}^{TA}\left(X;t\right) & =\frac{1}{q-1}\left[1-\frac{1}{{\left(\delta_{\left(0,t\right)}^{F}\right)}^{q-1}}\int_{0}^{t}{\left[\frac{f(x)}{F(t)}\right]}^{q}dx\right] \end{array}$$

(66) $$\begin{array}{cc} \; & =\frac{1}{q-1}\left\{1-\frac{1}{F(t)}E\left[{\left[\frac{f(X)}{F\left(t\right)\delta_{\left(0,t\right)}^{F}}\right]}^{q-1}|0<X<t\right]\right\}. \end{array}$$

where $\delta_{\left(0,t\right)}^{F}=\frac{1}{F(t)}\sup\left\{f\left(x\right)|x\in \left(0,t\right)\right\}$.

We notice that a similar relationship to (54) can be written for residual Tsallis entropies and for past Tsallis entropies:

(67) $$H^{TA}\left(X;t\right)={\left(\delta_{\left(t,\infty \right)}^{\bar{F}}\right)}^{1-q}H^{T}\left(X;t\right)+\log_{q}\delta_{\left(t,\infty \right)}^{\bar{F}},$$

(68) $${\bar{H}}^{TA}\left(X;t\right)={\left(\delta_{\left(0,t\right)}^{F}\right)}^{1-q}{\bar{H}}^{T}\left(X;t\right)+\log_{q}\delta_{\left(0,t\right)}^{F},$$

and the following theorem can be proven:

**Theorem** **5.**  *Residual Tsallis–Awad entropy for the concomitant of the r-th GOS from the FGM family is:*

(69) HTA(Y[r]*;t)=(δ(t,∞)G¯[r])1−qq−1{1−1G¯[r](t)q∑k=0q∑s=0k(−1)sqkks2k−sαkCr*k××EUfY(FY−1(U))q−1Uk−s|U>FY(t)}+logqδ(t,∞)G¯[r],

*where U is an U(0,1) random variable, and $\delta_{\left(t,\infty \right)}^{{\bar{G}}_{\left[r\right]}}=\frac{1}{{\bar{G}}_{\left[r\right]}\left(t\right)}\sup\left\{g_{\left[r\right]}\left(x\right)|x\in \left(t,+\infty \right)\right\}$.*

*Past Tsallis–Awad entropy for the concomitant of the r-th GOS from the FGM family is:*

(70) H¯TA(Y[r]*;t)=(δ(0,t)G[r](t))1−qq−1{1−1G[r](t)q∑k=0q∑s=0k(−1)sqkks2k−sαkCr*k××EUfY(FY−1(U))q−1Uk−s|U<FY(t)}+logqδ(0,t)G[r],

*where U is an U(0,1) random variable, and $\delta_{\left(0,t\right)}^{G_{\left[r\right]}}=\frac{1}{G_{\left[r\right]}}\sup\left\{g_{\left[r\right]}\left(x\right)|x\in \left(0,t\right)\right\}$.*

**Corollary** **5.**  *The residual Tsallis–Awad entropy for the concomitant of r-th-order statistic is:*

(71) HTA(Y[r];t)=(δ(t,∞)G¯[r])1−qq−1{1−1G¯[r](t)q∑k=0q∑s=0k(−1)sqkks2k−sαk(−1)kn−2r+1n+1k××EUfY(FY−1(U))q−1Uk−s|U>FY(t)}+logqδ(t,∞)G¯[r].

*The residual Tsallis–Awad entropy for the concomitant of r-th record value is:*

(72) HTA(Y[r];t)=(δ(t,∞)G¯[r])1−qq−1{1−1G¯[r](t)q∑k=0q∑s=0k(−1)sqkks2k−sαk(−1)k21−r−1k××EUfY(FY−1(U))q−1Uk−s|U>FY(t)}+logqδ(t,∞)G¯[r].

*In the case of progressive type II censoring order statistics with equi-balanced censoring scheme, the residual Tsallis entropy of the concomitant of r-th-order statistic from the FGM family is (59) with m=R, the removal number.*

*Similar results can be obtained for past Tsallis entropy.*

### 4.3. Fisher–Tsallis Information Number

Various generalizations of FIN have been proposed, see, for example, [53,54,55]. In [53], the FIN is generalized, replacing the expectation and the logarithm functions with their *q* variants, and in [54], a (β,q)-Fisher information is defined. We here consider the following extension FIN, which we call **Fisher–Tsallis information number**:

(73) $$I_{f}=E_{f}\left[{\left(\frac{\partial }{\partial x}\log_{q}f\left(x\right)\right)}^{2}\right],$$

where $\log_{q}$ is given by (30). This extension is a type of extension from [54], with β=2 and q=1.

For the concomitants of the GOS from FGM family, we have the following theorem which can be seen as an extension of the results obtained in [40]:

**Theorem** **6.**  For the r-th concomitant $Y_{\left[r\right]}^{*}$ of GOS from a FGM family, the Tsallis–Fisher information number for a location parameter is:

(74) $$I_{g_{\left[r\right]}}=I_{1}+I_{2}+I_{3},$$

*where*

$$\begin{array}{cc} I_{1} & =E_{U}\left[{\left(f_{Y}\left(F^{-1}\left(U\right)\right)\right)}^{-2q}{\left(f_{Y}^{\prime }\left(F_{Y}^{-1}\left(U\right)\right)\right)}^{2}{\left(1+C_{r}^{*}\alpha \left(1-2U\right)\right)}^{2-2q}\right],\\ I_{2} & =4C_{r}^{*2}\alpha^{2}\cdot E_{U}\left[{\left(f_{Y}\left(F^{-1}\left(U\right)\right)\right)}^{4-2q}{\left(1+C_{r}^{*}\alpha \left(1-2U\right)\right)}^{-2q}\right],\\ I_{3} & =-4C_{r}^{*2}\alpha \cdot E_{U}\left[f_{Y}\left(F^{-1}\left(U\right)\right){)}^{2-2q}f_{Y}^{\prime }\left(F_{Y}^{-1}\left(U\right)\right){\left(1+C_{r}^{*}\alpha \left(1-2U\right)\right)}^{1-2q}\right]. \end{array}$$

**Proof.**  From (73), we have

$$I_{g_{\left[r\right]}}=E_{g_{\left[r\right]}}\left[{\left(g_{\left[r\right]}\left(Y\right)\right)}^{-2q}{\left(g_{\left[r\right]}^{\prime }\left(Y\right)\right)}^{2}\right].$$

Using the expression (7) for the density $g_{\left[r\right]}$, it results in

$$\begin{array}{cc} I_{g_{\left[r\right]}} & =E_{g\left[r\right]}[f_{Y}{\left(Y\right)}^{-2q}{\left[1+C_{r}^{*}\alpha \left(1-2F_{Y}\left(Y\right)\right)\right]}^{-2q}\left.{\left[f_{Y}^{\prime }\left(Y\right)\left(1+C_{r}^{*}\alpha \left(1-2F_{Y}\left(Y\right)\right)\right)-2{\left(f_{Y}\left(Y\right)\right)}^{2}C_{r}^{*}\alpha \right]}^{2}\right]. \end{array}$$

Thus,

$$\begin{array}{cc} I_{g_{\left[r\right]}}= & E_{g\left[r\right]}[{\left(f_{Y}\left(Y\right)\right)}^{-2q}{\left[1+C_{r}^{*}\alpha \left(1-2F_{Y}\left(Y\right)\right)\right]}^{-2q}\\ \; & \cdot [{\left(f_{Y}^{\prime }\left(Y\right)\right)}^{2}{\left(1+C_{r}^{*}\alpha \left(1-2F_{Y}\left(Y\right)\right)\right)}^{2}+4{\left(f_{Y}\left(Y\right)\right)}^{4}C_{r}^{*2}\alpha^{2}-\\ \; & -4f_{Y}^{\prime }\left(Y\right){\left(f_{Y}\left(Y\right)\right)}^{2}C_{r}^{*}\alpha \left(1+C_{r}^{*}\alpha \left(1-2F_{Y}\left(Y\right)\right)\right)]]. \end{array}$$

After some computations,

$$\begin{array}{cc} I_{g_{\left[r\right]}}= & E_{g_{\left[r\right]}}\left[{\left(f_{Y}\left(Y\right)\right)}^{-2q}{\left(f_{Y}^{\prime }\left(Y\right)\right)}^{2}{\left(1+C_{r}^{*}\alpha \left(1-2F_{Y}\left(Y\right)\right)\right)}^{2-2q}\right]+\\ \; & +4C_{r}^{*2}\alpha^{2}E_{g_{\left[r\right]}}\left[f_{Y}{\left(Y\right)}^{4-2q}{\left(1+C_{r}^{*}\alpha \left(1-2F_{Y}\left(Y\right)\right)\right)}^{-2q}\right]-\\ \; & -4C_{r}^{*}\alpha E_{g_{\left[r\right]}}\left[{\left(f_{Y}\left(Y\right)\right)}^{2-2q}f_{Y}^{\prime }\left(Y\right){\left(1+C_{r}^{*}\alpha \left(1-2F_{Y}\left(Y\right)\right)\right)}^{1-2q}\right]. \end{array}$$

After the transformation $U=F_{Y}\left(Y\right)$, with U∼U(0,1) we obtain (74). □

### 4.4. Tsallis Divergence

We consider **Tsallis divergence** for two densities, $f_{1}$ and $f_{2}$, as it is defined in [44]:

(75) $$TD\left(Z_{1},Z_{2}\right)=\int_{-\infty }^{+\infty }f_{1}\left(z\right)log_{q}\left(\frac{f_{2}\left(z\right)}{f_{1}\left(z\right)}\right)dz$$

that can also be expressed as:

(76) $$TD\left(Z_{1},Z_{2}\right)=\frac{1}{1-q}E_{f_{1}}\left[{\left(\frac{f_{1}\left(z\right)}{f_{2}\left(z\right)}\right)}^{q-1}-1\right].$$

We notice that this divergence is the divergence considered in [36], with $\phi \left(x\right)=\log_{q}\left(1/x\right)$, it is the divergence analyzed in [56], with k=1−q, and it is Tsallis Relative Entropy from [57].

When q→1, Tsallis entropy becomes Shannon entropy and also Tsallis divergence becomes Kullback–Leibler divergence (33). The next theorem generalizes the results from [23], computing Tsallis divergence for two concomitants of GOS from the FGM family.

**Theorem** **7.**  *Let $Y_{\left[r\right]}$ and $Y_{\left[s\right]}$ the r-th and the s-th concomitants of the GOS from the FGM family with the densities $g_{\left[r\right]}$ and $g_{\left[s\right]}$. Then, the Tsallis divergence of $g_{\left[s\right]}$ from $g_{\left[r\right]}$ has the following form:*

(77) $$TD\left(Y_{\left[r\right]},Y_{\left[s\right]}\right)=\frac{1}{1-q}\left[D_{1}-D_{2}-1\right],$$

*where*

$$D_{1}=\frac{1}{q(q+1)}{\left(\frac{C_{r}^{*}}{C_{r}^{*}-C_{s}^{*}}\right)}^{q-1}{\left(1+C_{r}^{*}\alpha \right)}^{q+1}F_{1}\left(q+1,q-1,q+1,-\frac{C_{s}^{*}\left(1+C_{r}^{*}\alpha \right)}{C_{r}^{*}-C_{s}^{*}}\right),$$

$$D_{2}=\frac{1}{q(q+1)}{\left(\frac{C_{r}^{*}}{C_{r}^{*}-C_{s}^{*}}\right)}^{q-1}{\left(1-C_{r}^{*}\alpha \right)}^{q+1}F_{1}\left(q+1,q-1,q+1,-\frac{C_{s}^{*}\left(1-C_{r}^{*}\alpha \right)}{C_{r}^{*}-C_{s}^{*}}\right),$$

*with $F_{1}$ being the hypergeometric function.*

**Proof.**  In (76), we replace $f_{1}$ and $f_{2}$ with the concomitants densities:

$$g_{\left[r\right]}\left(y\right)=f_{Y}\left(y\right)\left[1+C_{r}^{*}\alpha \left(2F_{y}\left(y\right)-1\right)\right],$$

$$g_{\left[s\right]}\left(y\right)=f_{Y}\left(y\right)\left[1+C_{s}^{*}\alpha \left(2F_{y}\left(y\right)-1\right)\right],$$

and we compute the expectation:

(78) $$\begin{array}{cc} E_{g_{\left[r\right]}}\left[{\left(\frac{g_{\left[r\right]}\left(Y\right)}{g_{\left[s\right]}\left(Y\right)}\right)}^{q-1}\right] & =\int_{0}^{\infty }g_{\left[r\right]}\left(y\right){\left(\frac{g_{\left[r\right]}\left(Y\right)}{g_{\left[s\right]}\left(Y\right)}\right)}^{q-1}dy\\ \; & =\int_{0}^{\infty }f_{Y}\left(y\right)\left[1+C_{r}^{*}\alpha \left(2F_{Y}\left(y\right)-1\right)\right]{\left(\frac{f_{Y}\left(y\right)\left[1+C_{r}^{*}\alpha \left(2F_{Y}\left(y\right)-1\right)\right]}{f_{Y}\left(y\right)\left[1+C_{s}^{*}\alpha \left(2F_{Y}\left(y\right)-1\right)\right]}\right)}^{q-1}dy. \end{array}$$

First, we make the transformation:

$$F_{Y}\left(y\right)=u,\;\;y=F_{Y}^{-1}\left(u\right),\;\;f_{Y}\left(y\right)dy=du,$$

and we obtain

(79) $$\begin{array}{cc} E_{g_{\left[r\right]}}\left[{\left(\frac{g_{\left[r\right]}\left(Y\right)}{g_{\left[s\right]}\left(Y\right)}\right)}^{q-1}\right] & =\int_{0}^{1}\frac{{\left[1+C_{r}^{*}\alpha \left(2u-1\right)\right]}^{q}}{{\left[1+C_{s}^{*}\alpha \left(2u-1\right)\right]}^{q-1}}du. \end{array}$$

Then, we make the transformation:

2u−1=v,u=(v+1)/2,2du=dv,

and we obtain:

(80) $$\begin{array}{cc} E_{g_{\left[r\right]}}\left[{\left(\frac{g_{\left[r\right]}\left(Y\right)}{g_{\left[s\right]}\left(Y\right)}\right)}^{q-1}\right] & =\frac{1}{2}\int_{-1}^{1}\frac{{\left[1+C_{r}^{*}\alpha v\right]}^{q}}{{\left[1+C_{s}^{*}\alpha v\right]}^{q-1}}dv. \end{array}$$

We use the general formula:

(81) $$\begin{array}{cc} \frac{1}{2}\int_{-1}^{1} & \frac{{\left(1+ax\right)}^{A}}{{\left(1+bx\right)}^{B}}dx=\\ \; & =\frac{1}{A(A+1)}\left\{{\left(1+a\right)}^{A+1}{\left(1+b\right)}^{-B}{\left[\frac{a(1+b)}{a-b}\right]}^{B}F_{1}\left(A+1,B,A+1,-\frac{b(1+a)}{a-b}\right)\right.-\\ \; & \left.-{\left(1-a\right)}^{A+1}{\left(1-b\right)}^{-B}{\left[\frac{a(1-b)}{a-b}\right]}^{B}F_{1}\left(A+1,B,A+1,-\frac{b(1-a)}{a-b}\right)\right\}, \end{array}$$

where $F_{1}$ is the hypergeometric function. It results in:

$$E_{g_{\left[r\right]}}\left[{\left(\frac{g_{\left[r\right]}\left(Y\right)}{g_{\left[s\right]}\left(Y\right)}\right)}^{q-1}\right]=D_{1}-D_{2}.$$

□

## 5. Conclusions

This paper is focused on information measures related to Shannon entropy, Tsallis entropy, Fisher information, and divergences for the concomitants of GOS from the FGM family. We review the literature on the mentioned information measures and we generalize existing results. The study of the concomitants, pairs of the order statistics in a sample from a bivariate distribution, ordered by one variate, could have applications in reliability, for example, in the analysis of the lifetime uncertainty of complex systems. For this reason, we also discuss residual and past versions of the entropies. Considering generalized order statistics (GOS) results in an increasing complexity of computations, but it gives a general form of the computed measures that can be applied for the concomitants of various order statistics.

## Data Availability

Not applicable.

## References

[1] David H.A. (1973). Concomitants of order statistics. Bull. Inst. Int. Statist..

[2] Bhattacharya P. (1974). Convergence of sample paths of normalized sums of induced order statistics. Ann. Stat..

[3] Bayramoglu I. (2013). Reliability and mean residual life of complex systems with two dependent components per element. IEEE Trans. Reliab..

[4] Ozkut M. (2021). The (*n* − *k* + 1)-out-of-*n* concomitant system having *m* subcomponents and its reliability. J. Comput. Appl. Math..

[5] Scaria J., Mohan S. (2021). Dependence concepts and Reliability application of concomitants of order statistics from the Morgenstern family. J. Stat. Theory Appl..

[6] Kamps U. (1995). A concept of generalized order statistics. J. Stat. Plan. Inference.

[7] Balakrishnan N., Lai C.D. (2009). Continuous Bivariate Distributions.

[8] Kotz S., Johnson N.L. (1977). Propriétés de dépendance des distributions itérées généralisées à deux variables Farlie-Gumbel-Morgenstern. C. R. Acad. Sci. Paris A.

[9] Huang J., Kotz S. (1984). Correlation structure in iterated Farlie-Gumbel-Morgenstern distributions. Biometrika.

[10] Huang J.S., Kotz S. (1999). Modifications of the Farlie-Gumbel-Morgenstern distributions. A tough hill to climb. Metrika.

[11] Bairamov I., Kotz S., Bekci M. (2001). New generalized Farlie-Gumbel-Morgenstern distributions and concomitants of order statistics. J. Appl. Stat..

[12] Bairamov I., Kotz S. (2002). Dependence structure and symmetry of Huang-Kotz FGM distributions and their extensions. Metrika.

[13] Aggarwala R., Balakrishnan N. (1996). Recurrence relations for single and product moments of progressive Type-II right censored order statistics from exponential and truncated exponential distributions. Ann. Inst. Stat. Math..

[14] Balakrishnan N., Cramer E. (2014). The Art of Progressive Censoring.

[15] Cramer E., Kamps U. (2003). Marginal distributions of sequential and generalized order statistics. Metrika.

[16] Farlie D.J.G. (1960). The performance of some correlation coefficients for a general bivariate distribution. Biometrika.

[17] Gumbel E. (1960). Bivariate exponential distributions. J. Am. Statist. Assoc..

[18] Morgenstern D. (1956). Einfache Beispiele Zweidimensionaler Verteilungen. Mitt. Math. Stat..

[19] Johnson N.L., Kotz S. (1977). On some generalized Farlie-Gumbel-Morgenstern distributions-II regression, correlation and further generalizations. Commun. Stat.-Theory Methods.

[20] Nelsen R.B. (2007). An Introduction to Copulas.

[21] Beg M., Ahsanullah M. (2008). Concomitants of generalized order statistics from Farlie–Gumbel–Morgenstern distributions. Stat. Methodol..

[22] Shannon C.E. (1948). A mathematical theory of communication. Bell Syst. Tech. J..

[23] Tahmasebi S., Behboodian J. (2012). Information properties for concomitants of order statistics in Farlie–Gumbel–Morgenstern (FGM) family. Commun. Stat.-Theory Methods.

[24] Tahmasebi S., Behboodian J. (2012). Shannon information for concomitants of generalized order statistics in Farlie–Gumbel–Morgenstern (FGM) family. Bull. Malays. Math. Sci. Soc..

[25] Awad A. (1987). A statistical information measure. Dirasat (Sci.).

[26] Ebrahimi N., Pellerey F. (1995). New partial ordering of survival functions based on the notion of uncertainty. J. Appl. Probab..

[27] Ebrahimi N. (1996). How to measure uncertainty in the residual life time distribution. Sankhyā Indian J. Stat. Ser. A.

[28] Di Crescenzo A., Longobardi M. (2002). Entropy-based measure of uncertainty in past lifetime distributions. J. Appl. Probab..

[29] Mohie EL-Din M.M., Amein M.M., Ali N.S.A., Mohamed M.S. (2015). Residual and past entropy for concomitants of ordered random variables of Morgenstern family. J. Probab. Stat..

[30] Havrda J., Charvát F. (1967). Quantification method of classification processes. Concept of structural *a*-entropy. Kybernetika.

[31] Tsallis C. (1988). Possible generalization of Boltzmann-Gibbs statistics. J. Stat. Phys..

[32] Hirica I.E., Pripoae C.L., Pripoae G.T., Preda V. (2022). Weighted Relative Group Entropies and Associated Fisher Metrics. Entropy.

[33] Wilk G., Włodarczyk Z. (2008). Example of a possible interpretation of Tsallis entropy. Phys. A Stat. Mech. Its Appl..

[34] Calì C., Longobardi M., Ahmadi J. (2017). Some properties of cumulative Tsallis entropy. Phys. A Stat. Mech. Its Appl..

[35] Paul J., Thomas P.Y. (2015). Tsallis entropy properties of record values. Calcutta Stat. Assoc. Bull..

[36] Nanda A.K., Paul P. (2006). Some results on generalized residual entropy. Inf. Sci..

[37] Zografos K. (2022). On reconsidering entropies and divergences and their cumulative counterparts: Csiszár’s, DPD’s and Fisher’s type cumulative and survival measures. Probab. Eng. Infor. Sci..

[38] Papaioannou T., Ferentinos K. (2005). On two forms of Fisher’s measure of information. Commun. Stat.-Theory Methods.

[39] Frieden B.R. (1998). Physics from Fisher information: A Unification.

[40] Tahmasebi S., Jafari A.A. (2013). Fisher information number for concomitants of generalized order statistics in Morgenstern family. J. Inform. Math. Sci..

[41] Barbu V.S., Karagrigoriou A., Preda V. (2017). Entropy and divergence rates for Markov chains: I. The Alpha-Gamma and Beta-Gamma case. Proc. Rom. Acad.-Ser. A.

[42] Barbu V.S., Karagrigoriou A., Preda V. (2018). Entropy and divergence rates for Markov chains: II. The weighted case. Proc. Rom. Acad.-Ser. A.

[43] Barbu V.S., Karagrigoriou A., Preda V. (2018). Entropy and divergence rates for Markov chains: III. The Cressie and Read case and applications. Proc. Rom. Acad.-Ser. A.

[44] Villmann T., Haase S. (2010). Mathematical Aspects of Divergence Based Vector Quantization Using Fréchet-Derivatives. Master’s Thesis.

[45] Póczos B., Schneider J. On the estimation of alpha-divergences. Proceedings of the Fourteenth International Conference on Artificial Intelligence and Statistics.

[46] Kullback S., Leibler R.A. (1951). On information and sufficiency. Ann. Math. Stat..

[47] Kullback S. (1959). Information Theory and Statistics.

[48] Furuichi S., Yanagi K., Kuriyama K. (2004). Fundamental properties of Tsallis relative entropy. J. Math. Phys..

[49] Furuichi S., Mitroi F.C. (2012). Mathematical inequalities for some divergences. Phys. A Stat. Mech. Its Appl..

[50] Vigelis R.F., De Andrade L.H., Cavalcante C.C. (2019). Properties of a generalized divergence related to Tsallis generalized divergence. IEEE Trans. Inf. Theory.

[51] Awad A., Alawneh A. (1987). Application of entropy to a life-time model. IMA J. Math. Control Inf..

[52] Sfetcu R.C., Sfetcu S.C., Preda V. (2021). Ordering Awad–Varma Entropy and Applications to Some Stochastic Models. Mathematics.

[53] Furuichi S. (2009). On the maximum entropy principle and the minimization of the Fisher information in Tsallis statistics. J. Math. Phys..

[54] Bercher J.F. (2013). Some properties of generalized Fisher information in the context of nonextensive thermostatistics. Phys. A Stat. Mech. Its Appl..

[55] Kharazmi O., Balakrishnan N., Jamali H. (2022). Cumulative Residual *q*-Fisher Information and Jensen-Cumulative Residual χ^2^ Divergence Measures. Entropy.

[56] Sfetcu R.C., Sfetcu S.C., Preda V. (2022). On Tsallis and Kaniadakis Divergences. Math. Phys. Anal. Geom..

[57] Koukoumis C., Karagrigoriou A. (2021). On entropy-type measures and divergences with applications in engineering, management and applied sciences. Int. J. Math. Eng. Manag. Sci..

